# PKM2 Determines Myofiber Hypertrophy In Vitro and Increases in Response to Resistance Exercise in Human Skeletal Muscle

**DOI:** 10.3390/ijms21197062

**Published:** 2020-09-25

**Authors:** Sander A. J. Verbrugge, Sebastian Gehlert, Lian E. M. Stadhouders, Daniel Jacko, Thorben Aussieker, Gerard M. J. de Wit, Ilse S. P. Vogel, Carla Offringa, Martin Schönfelder, Richard T. Jaspers, Henning Wackerhage

**Affiliations:** 1 Department for Sport and Health Sciences, Technical University of Munich, Georg-Brauchle-Ring 60/62, 80992 München/Munich, Germany; sander.verbrugge@tum.de (S.A.J.V.); martin.schoenfelder@tum.de (M.S.); 2 Department for the Biosciences of Sports, Institute of Sports Science, University of Hildesheim, Universitätsplatz 1, 31141 Hildesheim, Germany; 3 Department for Molecular and Cellular Sports Medicine, German Sport University Cologne, 50933 Cologne, Germany; d.jacko@dshs-koeln.de (D.J.); ausska@gmx.de (T.A.); 4 Laboratory for Myology, Department of Human Movement Sciences, Faculty of Behavioural and Movement Sciences, Vrije Universiteit Amsterdam, Amsterdam Movement Sciences, De Boelelaan 1108, 1081 HZ Amsterdam, The Netherlands; lianstadhouders@hotmail.com (L.E.M.S.); g.m.j.de.wit@vu.nl (G.M.J.d.W.); ilsevogel@gmail.com (I.S.P.V.); c.offringa@vu.nl (C.O.)

**Keywords:** Warburg effect, pyruvate kinase, insulin-like growth factor 1, glycolysis, skeletal muscle, hypertrophy, resistance exercise, metabolic reprogramming, cancer

## Abstract

Nearly 100 years ago, Otto Warburg investigated the metabolism of growing tissues and discovered that tumors reprogram their metabolism. It is poorly understood whether and how hypertrophying muscle, another growing tissue, reprograms its metabolism too. Here, we studied pyruvate kinase muscle (PKM), which can be spliced into two isoforms (PKM1, PKM2). This is of interest, because PKM2 redirects glycolytic flux towards biosynthetic pathways, which might contribute to muscle hypertrophy too. We first investigated whether resistance exercise changes PKM isoform expression in growing human skeletal muscle and found that PKM2 abundance increases after six weeks of resistance training, whereas PKM1 decreases. Second, we determined that *Pkm2* expression is higher in fast compared to slow fiber types in rat skeletal muscle. Third, by inducing hypertrophy in differentiated C2C12 cells and by selectively silencing *Pkm1* and/or *Pkm2* with siRNA, we found that PKM2 limits myotube growth. We conclude that PKM2 contributes to hypertrophy in C2C12 myotubes and indicates a changed metabolic environment within hypertrophying human skeletal muscle fibers. PKM2 is preferentially expressed in fast muscle fibers and may partly contribute to the increased potential for hypertrophy in fast fibers.

## 1. Introduction

With age, we lose skeletal muscle mass and force, which impacts on daily activities and generally decreases quality of life [1]. Sufficient muscle mass is also important for the prevention of obesity and type 2 diabetes, and thus metabolic health, and improves the chances of surviving critical illness or severe trauma [2]. Muscle mass is a product of the number of muscle fibers as well as their cross-sectional area. Resistance exercise stimulates muscle protein synthesis which maintains or increases muscle mass [3]. Key candidate resistance exercise stimuli are mechanical load, metabolic stress and exercise-induced muscle damage [4] known to activate the serine/threonine kinase mTOR within the mTORC1 complex [5]. This is an important step because mTORC1 is the major but not sole regulator of muscle protein synthesis [6]. 

In adult human skeletal muscle, three different muscle fiber types (i.e., type I, type IIa and type IIx) are distinguished which are characterized by the myosin heavy chain isoform that is expressed (Myh7, Myh2 or Myh1) [7]. Interestingly, type I fibers are relatively small with a high oxidative- but also a higher protein synthesis capacity than type II fibers [8], while intriguingly type II fibers show more hypertrophy than type I fibers after resistance exercise [9,10]. Because type II fibers rely more on glycolysis for energy production than type I fibers [7], there might be a yet an unknown role of glycolysis in skeletal muscle hypertrophy that could explain the distinct growth responses seen in type I and type II fibers. 

Cancer and muscle hypertrophy are both situations where biomass increases depend on mTORC1 and other anabolic signaling processes [6,11]. In cancer cells, growth is additionally accompanied by a metabolic reprogramming which is acknowledged as one of the hallmarks of cancer [12]. Metabolic reprogramming of cancer was first discovered by Otto Warburg and co-workers who found that tumors take up more glucose and synthesize more lactate than non-growing cells and organs, even when oxygen is present [13,14]. The purpose of the metabolic reprogramming in cancer was poorly understood for many decades. However, today we know that a key function of such metabolic reprogramming is to generate glycolytic intermediates and other molecules as substrates for anabolic reactions such as nucleotide → RNA/DNA and amino acid → protein synthesis [15]. The energy metabolism and anabolic pathways that are reprogrammed vary between different types of cancer [16]. 

In the muscle hypertrophy literature, as well as in our own research, we found evidence that a hypertrophying skeletal muscle reprograms its metabolism in a similar manner to cancer cells. For example, growing muscles take up more glucose over several days after a bout of resistance exercise [17,18]. Furthermore, the muscle growth factor IGF-1 increases lactate dehydrogenase expression and glycolytic flux in hypertrophying C2C12 myotubes [19]. On the other hand, blocking glycolysis abolishes myotube growth (Stadhouders et al., 2020, submitted), which also indicates that hypertrophying muscle cells reprogram their metabolism during hypertrophy. We therefore decided to investigate whether metabolic enzymes that are involved in metabolic reprogramming in cancer also contribute to myotube growth in vitro and alter their abundance in human skeletal muscle under growth stimulation by resistance exercise. 

In cancer, a key cancer metabolic reprogramming-associated enzyme is pyruvate kinase muscle (HGNC gene symbol: *PKM*; EC 2.7.1.40). PKM normally catalyzes the last step of glycolysis:

Phosphoenolpyruvate + ADP ↔ pyruvate + ATP. 

However, the *PKM* gene is alternatively spliced as either *Pkm1* or *Pkm2*. Even though “muscle” is part of its name, PKM and especially the PKM2 isoform have been much more investigated in cancer than in muscle. PKM1 includes the amino acids encoded by exon 9 but not those encoded by exon 10, whereas PKM2 includes exon 10 but not exon 9 [20,21]. The splicing of PKM is regulated by the cancer gene *Myc* and by heterogeneous nuclear ribonucleoproteins (hnRNPs) [22]. PKM2 has different functions than PKM1. PKM1 is a constitutively active enzyme in the cytosol generating pyruvate, whereas PKM2 is less enzymatically active and can translocate to the nucleus where it contributes to the metabolic reprograming in cancer and the accretion of biomass [23,24]. PKM1 and PKM2 normally occur as a tetramer but PKM2 can also exist as a dimer that is less catalytically active and that has non-glycolytic, regulatory functions [23]. While a low PKM2 activity promotes conversion of pyruvate to lactate, a high activity of both PKM2 and PKM1 promotes conversion to acetyl-CoA and fuels oxidative metabolism. PKM2 exists as either a low-activity dimer or a high-activity tetramer [25]. Cancer cells predominantly express the low-activity dimer form of PKM2, which is associated with enhanced lactate production [23,26,27]. Cytoplasmic PKM acts as an enzyme promoting the production of glycolytic intermediates and biosynthesis of nucleotides and amino acids. The dimeric PKM2 also translocates to the nucleus and this nuclear fraction regulates transcription of genes involved in cell proliferation, glycolysis and also PKM2 itself [28,29].

Interestingly, alternative PKM splicing also occurs in the skeletal muscle lineage. For example, embryonal muscle expresses PKM2, which helps to regulate proliferation [30] and then shifts to PKM1 in adult muscle [31]. Similarly, C2C12 myoblasts change from PKM2 to PKM1 expression when differentiating into myotubes [22]. Finally, PKM2 is expressed in some human rhabdomyosarcomas [21], upregulated in the skeletal muscle of type 2 diabetes mellitus patients [32] and in myotonic dystrophy type 1 [31]. 

Given that PKM splice variants typically seem to shift from PKM2 in a growing, developing muscle to PKM1 in a non-growing adult muscle, we wanted to find out whether the stimulation of muscle hypertrophy increases PKM2 protein abundance and whether PKM2 limits muscle size. To test this hypothesis, we conducted a combined human muscle biopsy study where we measured PKM isoforms and cell culture experiments, where we selectively knocked down PKM1 and/or PKM2 isoforms in C2C12 myotubes. These experiments reveal that PKM2 protein is more abundant in more glycolytic type II fibers which have a greater capacity for hypertrophy than type I fibers [8]. We also found that PKM2 protein abundance increases in human vastus lateralis after six weeks of resistance training. In cultured C2C12 myotubes, the loss of PKM2 but not PKM1 reduces the muscle size of control and IGF-1-hypertrophy-stimulated myotubes, suggesting that PKM2 contributes to muscle hypertrophy. Collectively, this identifies PKM2 as a resistance exercise-regulated glycolytic enzyme that changes isoform expression in response to hypertrophy in human skeletal muscle and critically regulates hypertrophy in muscle cells. This also provides further evidence that a hypertrophying muscle reprograms its metabolism with some parallels to cancer. 

## 2. Results

**Figure 1:**
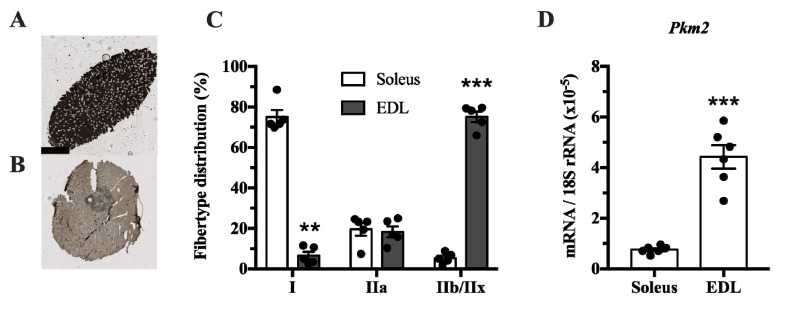
*Pkm2* expression is highest in extensor digitorum longus (EDL) rat muscle that predominantly consists of type 2 fibers. (**A**,**B**) ATPase staining of (**A**) soleus and (**B**) EDL rat muscle to determine fiber types (*n* = 4). (**C**) Fiber type distribution of rat soleus and EDL muscle. (**D**) Pkm2 mRNA expression in rat soleus and EDL (*n* = 6). Black circles indicate individual data points. * Significantly different from control, unpaired *t*-test and Mann–Whitney U test (*p* < 0.05); ** (*p* < 0.01); *** (*p* < 0.001). Scale bar in **A** indicates 1000 µm.

The muscle isoform of pyruvate kinase can be differentially spliced into a PKM1 isoform where exon 9 is expressed, and a PKM2 isoform where exon 10 is expressed. This isoform is mainly expressed in proliferating myoblasts [22]. Since pyruvate kinase is involved in glycolysis, we investigated whether *Pkm2* expression differs between glycolytic and predominantly oxidative muscle. We compared the soleus, which has mainly oxidative type I fibers, with extensor digitorum longus (EDL) rat muscle, which has a majority of glycolytic type IIx fibers (Figure 1A–C). This revealed that *Pkm2* expression is about five times higher in glycolytic EDL muscle than in the soleus (Figure 1D). 

**Figure 2:**
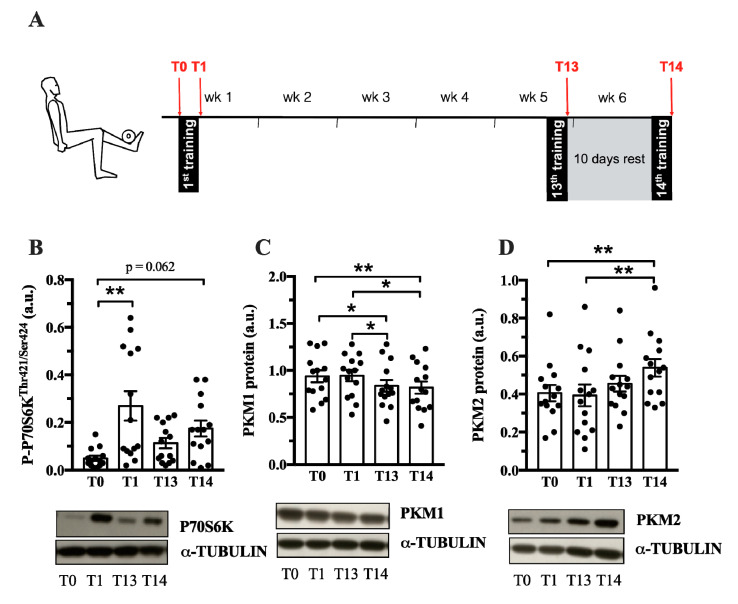
PKM2 and PKM1 abundance in vastus lateralis during 6 weeks of resistance training. (**A**) During 6 weeks of resistance exercise, muscle biopsies from vastus lateralis where collected at 4 different time points (red arrows): at rest (T0), after the 1st training session (T1), after the 13th training session (T13) and the 14th training session. Protein abundance of P-P70S6K (**B**), PKM1 (**C)** and PKM2 (**D**) (*n* = 14). Protein normalized to α-Tubulin. Black circles indicate individual data points. * Significantly different between indicated conditions, repeated measures ANOVA with Bonferroni post hoc test, or Friedman’s test with Dunn’s post hoc test (*p* < 0.05); ** (*p* < 0.01); *** (*p* < 0.001).

*Pkm2* expression is associated with both physiological and pathological growth processes, e.g., during development, regeneration or cancer. Until now, it is not known whether PKM2 plays a role in muscle hypertrophy induced by resistance training. Therefore, we investigated whether acute human resistance exercise (Exercise protocol in Figure 2A) or a period of resistance training affects PKM2 and PKM1 protein abundance. As expected, resistance training increased myofiber size of type I and type IIa/x fibers (Figure S1), as well as P70S6K and AKT phosphorylation (Figure 2B, Figure S2A), indicating activation of protein synthesis as a cause of gains in muscle mass [33]. PKM2 protein levels did not differ after one bout of resistance exercise (Figure 2D); however, a six-week training period of resistance exercise increased protein abundance (Figure 2D). In contrast, over the same time course, PFK1 and PKM1 levels decreased (Figure S2B and Figure 2C). This could suggest that *PKM* is preferentially spliced to the M2 isoform when muscle protein synthesis increases in response to resistance training. GAPDH remained unaltered by chronic training (Figure S2C).

**Figure 3:**
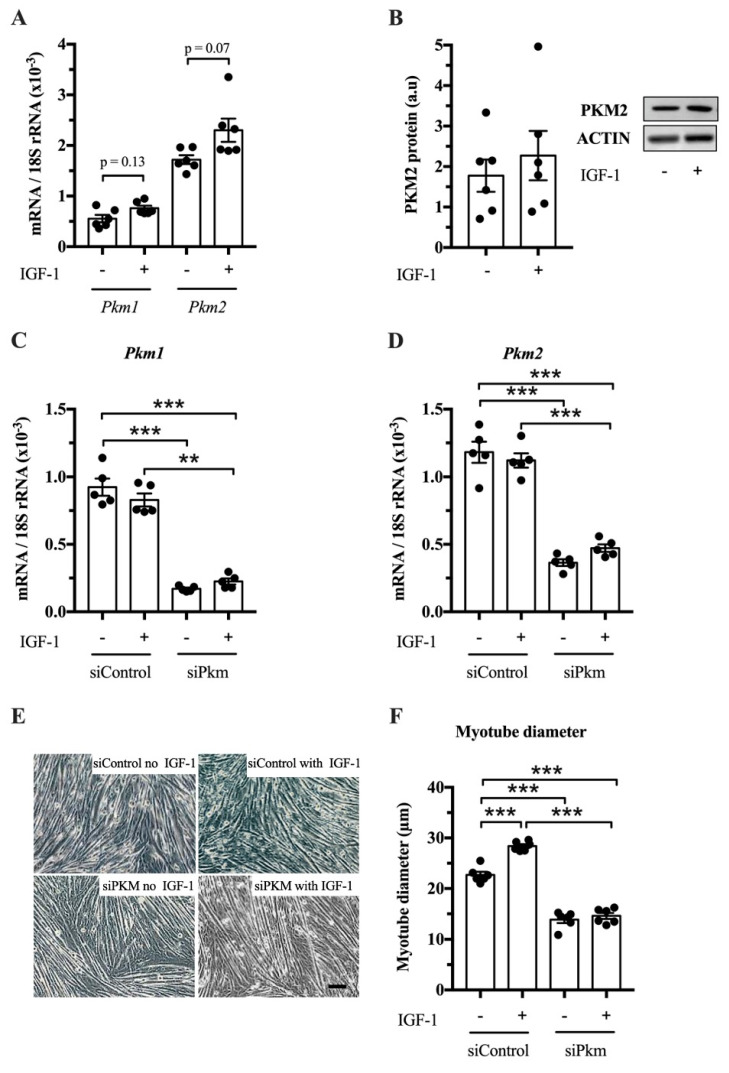
Effect of joint knock down of *Pkm1* and of *Pkm2* on C2C12 myotube size. (**A**) *Pkm1* and *Pkm2* mRNA in C2C12 myotubes following 24 h IGF-1 treatment. (**B**) PKM2 protein abundance in IGF-1 treated C2C12 myotubes. (**C**,**D**) *Pkm1* and *Pkm2* mRNA expression following siRNA interference of *Pkm* (*n* = 5). (**E**,**F**) C2C12 myotube diameter (*n* = 6). Black circles indiciate individual data points. Scale bar is 100 μm. * Significantly different between indicated conditions, unpaired *t*-test, Mann–Whitney U test or two-way ANOVA with Bonferroni post hoc test (*p* < 0.05); ** (*p* < 0.01); *** (*p* < 0.001).

After establishing that PKM1 and PKM2 abundance changes in response to resistance exercise, we studied whether PKM splicing is regulated by IGF-1 in vitro and whether PKM1, PKM2 or both limit muscle size. IGF-1 treatment tends to increase *Pkm1* and *Pkm2* mRNA in C2C12 myotubes (*p* = 0.13 and *p* = 0.07, respectively) (Figure 3A), but not clearly PKM2 protein abundance (Figure 3B). To investigate whether PKM plays a role in hypertrophy, we knocked down total *Pkm* by siRNA while stimulating hypertrophy with IGF-1. Interference of total *Pkm* expression reduced *Pkm1* mRNA, under basal and IGF-1-stimulated conditions by 82% and 73%, respectively. *Pkm2* mRNA was reduced by 69% and 58%, respectively (Figure 3C,D). Consequently, *Pkm* knock down reduced myotube size by 39% and 49%, under basal conditions and after IGF-1 stimulation, respectively (Figure 3E,F). This suggests that either *Pkm1* or *Pkm2* or both are required for normal myotube size. 

**Figure 4:**
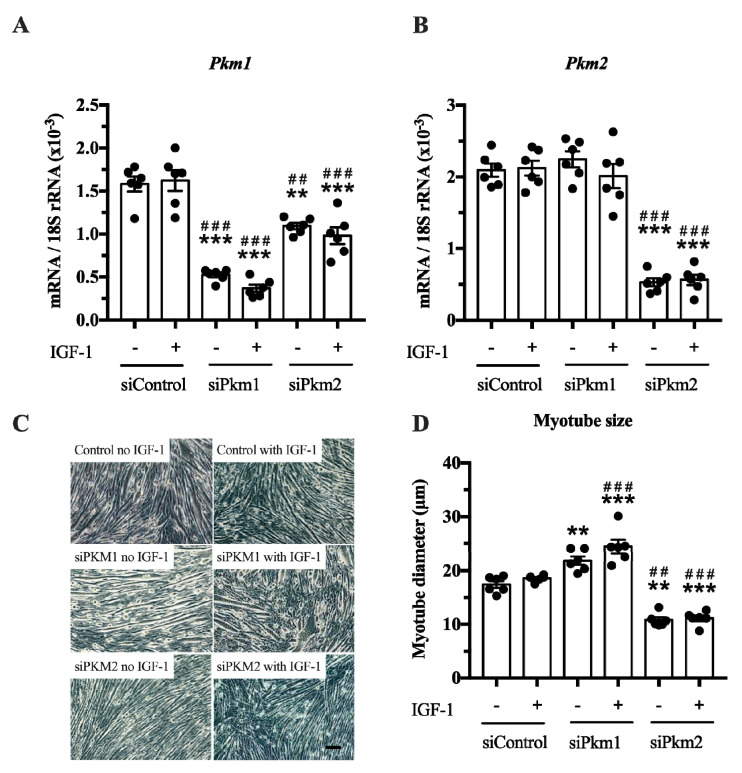
Effect of *Pkm1* or *Pkm2* knock down on C2C12 myotube size. (**A**,**B**) *Pkm1* and *Pkm2* mRNA expression following siRNA for *Pkm1* and *Pkm2* (*n* = 5). (**C**,**D**) C2C12 myotube diameter after siRNA treatment for *Pkm1* or *Pkm2* (*n* = 6). Scale bar is 100 μm. Black circles indicate individual data points. * Significantly different to unstimulated siControl (*p* < 0.05); ** (*p* < 0.01); *** (*p* < 0.001)., **#** significantly different to IGF-1 stimulated siControl (*p* < 0.05); ## (*p* < 0.01); ### (*p* < 0.001), Mann–Whitney U test or two-way ANOVA with Bonferroni post hoc test.

In the second experiment, we selectively knocked down either *Pkm2* or *Pkm1* (Figure 4A,B). This experiment revealed that only the knock down of *Pkm2*, but not that of *Pkm1*, reduced C2C12 myotube size by 38% and 40%, under basal and IGF-1-stimulated conditions, respectively (Figure 4C,D). In contrast, the knock down of *Pkm1* alone increased C2C12 myotube size by 25 and 30% in control and IGF-1-stimulated myotubes, respectively (Figure 4D), suggesting that PKM1 negatively regulates myotube size. Note that although interference of *Pkm2* decreases *Pkm1* mRNA, this does not increase myotube size. This indicates that the growth-reducing effect of *Pkm2* knock down is larger than the growth-promoting effect of *Pkm1* knock down (Figure 4A,D). Together, this suggests that PKM2 is the critical, muscle-size limiting PKM isoform. 

## 3. Discussion

In cancer, an altered regulation of glycolytic enzymes including PKM2 helps to reprogram metabolism so that glycolytic intermediates and other metabolites are increasingly channeled into anabolic reactions that support the accretion of biomass [15]. Here, we show evidence for a similar metabolic reprogramming in hypertrophying skeletal muscle. Specifically, we report that *Pkm2* is more expressed in type II than type I muscle fibers, that resistance training causes a small, delayed increase in PKM2 abundance in human skeletal muscle and that PKM2 limits muscle hypertrophy. 

Our first observation is that the *Pkm2* splice variant mRNA and/or protein was detected in rat soleus, EDL and human vastus lateralis (Figure 1 and Figure 2). This is in line with earlier observations that *Pkm2* mRNA is detected in embryonal muscle [31], C2C12 myoblasts [22] and rhabdomyosarcomas [21]. Collectively, this confirms that *Pkm2* is indeed expressed in skeletal muscle as a splice variant of the *PKM* gene. Because these analyses were performed on whole skeletal muscle, we cannot say in what fiber types or other cells, such as satellite cells, fibroblasts or endothelial cells, it is expressed [34].

Our data show that the PKM2 protein abundance increases in whole muscle after a six-week period of resistance training (Figure 2). One bout of resistance exercise did not alter PKM2 levels, as previously reported [35]. It takes 13–14 training sessions to increase PKM2 abundance (Figure 2C), which coincides with AKT and P70S6K phosphorylation (Figure 2A, Figure S2A). Interestingly, AKT has been shown to bind and phosphorylate PKM2 in cancer cells [36,37], suggesting that there may be other mechanisms of PKM2 regulation in muscle. Notably, PKM2 was higher following the 10-day rest period (T14) compared to levels after 13 training sessions (Figure 2D). A detraining phase following resistance exercise can increase the number of type IIx fibers, creating a more glycolytic muscle [9,38]. Perhaps this could explain the increase in PKM2 abundance after the rest period. However, other glycolytic enzymes, GAPDH, PFK1 and PKM1 do not follow this pattern (Figure S2, Figure 2 C). 

In contrast to PKM2 (Figure 2D), PKM1 abundance decreases after six weeks of resistance training (Figure 2C). PKM1 and PKM2 are encoded by the same gene and are differentially expressed by alternative splicing [39]. This suggests that a resistance exercise-associated mechanism affects alternative splicing in favor of the PKM2 isoform, decreasing PKM1 abundance. Interestingly, MYC is a regulator of *PKM* splicing [22] and MYC expression increases after acute resistance exercise (Metamex.eu; [40]) suggesting a possible mechanism by which PKM splicing is regulated by resistance training.

PKM2 function can additionally be regulated by post-translational modifications, such as phosphorylation, acetylation and glycosylation [25], and we did not measure whether such regulation took place in resistance-trained muscle. For example, the muscle size regulating kinase AKT phosphorylates PKM2 [36]. Furthermore, a 1 h bout of electrically evoked maximal-intensity muscle contractions phosphorylates PKM at multiple sites [41]. Allosteric binding of metabolites to PKM2 has also been shown to alter PKM2 activity. Metabolites including serine, SAICAR, pyruvate, phenylalanine, alanine, ATP, and thyroid hormone have the ability to regulate PKM2 [25]. The concentrations of these metabolites are also altered by resistance exercise [42]. Thus, PKM2 activity may be regulated through various mechanisms in a muscle during and after resistance exercise. 

In addition to resistance training, there are other situations where PKM splicing may play a role in muscle. For example, PKM2 has been implicated in muscle wasting and type 2 diabetes mellitus [32,43]. Patients with myotonic dystrophy type 1 show selectively increased PKM2 levels in type I fibers, which are most susceptible for atrophy in this disease [43]. In addition, skeletal muscle from type 2 diabetes subjects shows a higher expression of PKM2 [32]. PKM2 is regulated by reactive oxygen species (ROS) that play a role in diabetes [44] and contribute to muscle atrophy [45]. On the other hand, ROS and hypoxia are implicated in muscle hypertrophy [46,47]. Low-intensity resistance exercise in a hypoxic environment or training under blood flow restriction induces muscle hypertrophy [46]. Blood flow restriction increases lactate concentration [48], indicating increased glycolytic activity. Moreover, the percentage of type II fibers increases at the expense of type I fibers under hypoxic conditions in mice [48]. Together, these changes are indicative of a more glycolytic phenotype that could support the synthesis of amino acids and contribute to hypertrophy. Future research should elucidate whether PKM splicing is involved in these phenomena.

In this study, we found that the loss of *Pkm* (Figure 3) and specifically the loss of *Pkm2*, but not of *Pkm1* (Figure 4), reduces C2C12 myotube size. This fits the general observation that PKM2 contributes to cell and tissue growth in different contexts such as development, regeneration and cancer [20,23,24,27]. A limitation of the study is, however, that we had no appropriate animal model available where the long-term effects of hypertrophy models in PKM-isoform-specific mice would provide the possibility to study long term physiological and phenotypic effects. *Pkm2* knock-out mice are viable and do not show morphological differences in skeletal muscle [49]. These mice, however, compensate for a loss of *Pkm2* by upregulating *Pkm1* expression during embryogenesis. In addition, the researchers did not study whether a loss of Pkm2 would limit skeletal muscle hypertrophy. Future studies should seek to verify the role of PKM2 in muscle mass regulation in inducible *Pkm2*-specific knockout mice or by using PKM2 inhibitors in muscle hypertrophy models in vivo [50].

While PKM2 loss reduced myotube size, knock down of PKM1 was associated with an increase in myotube diameter and suggests an inhibitory role on myofiber growth at least in myotubes via an unknown mechanism. We also recognized in resistance exercise-stimulated and hypertrophying human skeletal muscle that PKM1 decreases while PKM2 increases. Hence, the reduced expression of PKM1 in concert with increased PKM2 expression may contribute to a fine tuning of the cytoplasm-muscle fiber environment to support myofiber hypertrophy in adult muscle. We acknowledge that a direct comparison of myotube growth of differentiated C2C12 myotubes and adult muscle fibers subjected to resistance exercise is limited. Mechanical strain of human muscle fibers activates signaling pathways that regulate protein synthesis [4,6,51], while IGF-1 plays a subtler role in growth responses and its role is still being discussed [52]. This situation is different in myotubes, where, in our approach, IGF-1 was used for growth activation. Because IGF-1 itself stimulates glycolytic flux and therefore lactate production [19], this might have altered responses in myotubes to an unknown extent compared to human myofibers.

As a limitation of our approach, we also have to acknowledge that we were not able to differentiate whether PKM isoforms differed in their activity in dependency of the applied condition in C2C12 cells or in human skeletal muscle between rest and post-resistance exercise. As introduced, the expressed isoform, as well as a shift from the tetrameric to dimeric state and vice versa, regulates the activity and nuclear translocation of PKM enzymes [25,28]. The dimeric form of PKM2 has a lower activity and is predominantly expressed in cancer cells due to mTOR activation. Because mTOR related signaling is increased by resistance exercise [51,53] and IGF-1 [11], it is conceivable that in our approach, the experimental conditions may likely have enhanced the expression of the dimeric isoform.

In future approaches it has to be specifically investigated, under which time course and conditions, tetrameric and dimeric forms of PKM will occur and how the nuclear localization of PKM impacts gene expression patterns in exercising muscle. It will also be of crucial interest to determine a potential role of PKM2 in co-regulating MyHC expression. We show that in fast mouse muscle, PKM2 levels are significantly higher than PKM1 levels. It is well known that the neural firing pattern is a dominant driver of pathways that are responsible for regulating the fiber type-specific gene expression and finally the fiber type (e.g., calcium signaling) [54]. Moreover, IGF-1 stimulates the expression of type IIb MyHC in myotubes [55].

It is questionable whether the gene set of an adult fast muscle fiber is per se programmed in a way that PKM2 levels are concomitantly upregulated with the shift towards a fast type or that the metabolic state of fast fibers is involved in determining the expression of PKM2. To the best of our knowledge, it has not been shown that PKM2 specifically binds to MyHC promoters of IIB fibers in rodents or IIX promotors in human skeletal muscle. Insights into the role of PKM2 in determining muscle fiber size and phenotype warrant further investigation.

Finally, more broadly, this study provides further evidence for the model that hypertrophying skeletal muscle reprograms its metabolism similar to cancer cells. This possibility has been discussed in the context of muscle regeneration, where satellite cell-derived myoblasts reprogram their metabolism when they proliferate [56,57,58] but not for muscle hypertrophy, where muscle fibers grow without proliferation. However, when a muscle hypertrophies, the ≈30% of mass that is not protein [59] needs to be synthesized, too, and also muscle fibers may synthesize non-essential amino acids as substrates for protein synthesis which may require metabolic reprograming. A specific example is ribosomal biogenesis which increases after resistance training [60]. Because ribosomes are comprised mainly of ribosomal RNA, this requires increased nucleotide → rRNA → ribosome synthesis. In relation to this, it has been shown that the first step, nucleotide production, is regulated by de novo nucleotide synthesis [61].

In summary, the PKM splice variant PKM2 increases after six weeks of resistance training in humans and limits myotube size in vitro. We conclude that augmented PKM2 expression is a contributing factor to support the cellular environment for muscle growth.

## 4. Materials and Methods

### 4.1. Male Human Biopsy Study

We used biopsy samples that were collected in a study that investigated the anabolic and autophagic response of skeletal muscle over the time course of six weeks of resistance exercise. Parts of this study were already published under different thematic aspects and details of the training regimen are described here [62]. Subjects were informed about the study orally and in text form in advance and gave their written informed consent prior to participation in the study. The local ethics committee of the German Sports University Cologne approved the study which was conducted following the guidelines of the declaration of Helsinki. Briefly, 14 physically active male sports students (24 ± 3 y, 183 ± 7 cm, 79 ± 0.9 kg) conducted thrice weekly resistance exercise for in sum 14 resistance training sessions. Muscle biopsies were collected at rest (T0), after the first (T1), after the 13th (T13) and, after 10 days of rest, the 14th resistance training session (T14). Prior to the study, subjects were tested for 10 repetitions maximum (10 RM) on a dual-legged knee-extension and leg press machine (Gym 80, Gelsenkirchen, Germany), where the training sessions were carried out. For all further training sessions (1–14), subjects performed three sets ranging between eight and twelve repetitions of leg extensions followed by leg press exercises. The movement speed consisted of 2 s concentric, 1 s isometric and 2 s eccentric movement with an 80 degree range of motion. We firstly chose to conduct a resting phase of 10 days to fully restore muscle regeneration before the last training session. Secondly, it has been reported that the application of a short period of detraining may lead to increased expression of glycolytic enzymes [38]. By doing this, we aimed to direct skeletal muscle adaptations as far as possible towards a glycolytic phenotype.

### 4.2. Muscle Biopsies

Muscle biopsies were collected from the mid-portion of the vastus lateralis muscle of one leg 45 min after the last repetition of leg press exercise. Muscle biopsies of vastus lateralis muscle were collected following local anesthesia using the Bergström biopsy method with additional suction [63]. Approximately 100 mg of muscle tissue was extracted, freed from blood and immediately frozen in liquid nitrogen. Twenty milligrams of muscle tissue were homogenized in ice-cold lysis buffer (Cell Signaling, Cat#9803, Beverly, MA, USA) using a commercially available micro-dismembrator (Braun, Melsungen, Germany). Homogenates were rotated for 60 min at 4 °C and centrifuged at 10,000× *g* for 10 min at 4 °C, before obtaining the supernatants which represent the sarcoplasmic protein fractions. The protein content of each sample was determined by the Lowry test kit (BioRad Laboratories GmbH, Cat#5000111, Munich, Germany). Homogenates of each subject were diluted to a protein concentration of 1.5 μg/μL homogenate. Homogenates of 25 μg protein were thawed on ice, suspended in buffer (0.5 M Tris–HCl, 10% glycerol, 2% sodium dodecyl sulphate, 5% 2-mercaptoethanol and 0.05% bromophenolblue) and heated at 95 °C for 7 min.

### 4.3. Western Blotting

Skeletal muscle lysates were diluted in 5 times Laemmli SDS buffer and denatured for 5 min at 95 °C, prior to western blotting. Samples were then electrophoresed on either 12% or 4–12% gradient Bis-Tris gels (Bio-rad, Cat#3450125, Hemel Hempstead, UK) with 3-morpholinopropane-1-sulfonic acid (MOPS) buffer, equilibrated in transferbuffer (25 M Tris, 192 M glycine, 20% ethanol, 0.0375 SDS) and transferred onto polyvinylidene difluoride (PVDF) membranes (GE Healthcare, Cat#15269894, Chicago, IL, USA) using a semi-dry transfer blotter (34 min, 1.2 mA, 25 V, Bio-rad). Membranes were blocked for 1 h with 5% milk powder dissolved in Tris buffered saline (TBS-T) containing 150 mM NaCl, 10 mM Tris-HCl; 1% Tween-20, SigmaAldrich, Cat#P1379, Steinheim, Germany; pH 7.6, then incubated with primary and secondary antibodies prior to fluorescent imaging. For human Western blots, the following primary antibodies were used: PKM1 (1:1500; Cell Signaling Technologies, Cat#7067, Danvers, MA, USA, RRID:AB_2715534), PKM2 (1:1500, Cell Signaling Technologies, Cat#4053, Danvers, MA, USA, RRID:AB_1904096), PFK (1:1500, Santa Cruz, Cat#sc-377346, Santa Cruz, CA, USA), phospho AKT Ser 473 (1:1500, Cell Signaling Technologies, Cat#4060, Danvers, MA, USA, RRID:AB_2315049), phospho P70 S6K Thr421/Ser424 (1:1500, Cell Signaling Technologies, Cat#9204, Danvers, MA, USA, RRID:AB_2265913). HRP conjugated secondary antibodies (1:8000, Cell Signaling Technologies, Cat#7074 (RRID:AB_2099233) rabbit and Cat#7076 (RRID:AB_330924) mouse, Danvers, MA, USA) were used and chemiluminescence development was conducted by adding Super Signal West Dura luminol agent onto the membrane (Thermo Fisher Scientific, Cat#34076, Northumberland, UK) following the manufacturer’s instructions. Densities of protein bands were normalized to α-TUBULIN (1:8000, DSHB, Cat#12G10, IOWA City, IOWA, USA, RRID:AB1157911) and quantified by densitometric analysis using ImageJ software (National Institutes of Health, USA, RRID:SCR_003070). 

### 4.4. Immunohistochemistry of Human Skeletal Muscle

Consecutive 7 µm cross-sections from each biopsy were cut with a LEICA CM7300 Cryostat and mounted on Polysine^®^ slides (VWR International GmbH, Cat#631-1349, Darmstadt, Germany) air-dried and stored at −80 °C until further analysis. For staining, slides were initially brought to room temperature and afterwards incubated for 8 min in −20 °C acetone. Sections were then blocked in 0.05 mM TBS containing 5% bovine serum albumin (BSA) for 1 h at room temperature. To detect type I and type II fibers and determine the borders of myofibers at the basement membrane, cross sections were incubated overnight at 4 °C with monoclonal mouse primary antibodies raised against human adult slow myosin heavy chain type I (1:200, DSHB Cat#A4.951, Iowa City, USA, RRID:AB_528385) and dystrophin (1:25, DSHB Cat#MANEX1011B(1C7), Iowa City, USA, RRID:AB_2618171). All primary antibodies were diluted in 0.8% BSA-TBS. The following morning, slides were rinsed 5 times for 5 min with TBS and then incubated for 1 h with goat-anti mouse polyclonal biotinylated secondary antibodies (1:400, Agilent Dako, Cat# E0432, Glostrup, Denmark, RRID:AB_2313609) diluted in 0.05 mM TBS. Slides were then incubated for 1 h with Streptavidin biotinylated Horseradish Peroxidase complex (1:400, Amersham Biosciences, Cat#RPN1051, Uppsala, Sweden) diluted in TBS. Staining was carried out using a 3,3′-diaminobenzidine (DAB) solution (0.09 M phosphate buffer (pH 7.4), 2.2 mM DAB, 7.03 mM ammonium chloride, 0.93 mM nickel sulfate, 10.44 mM ß-D-glucose and 0.000024 mM glucose oxidase). Hereafter, the staining procedure was repeated. Cross-sections were then incubated overnight with primary antibodies raised against type I and IIa fibers (1:100, DSHB Cat#N2.261, Iowa City, IA, USA, RRID:AB531790). All other procedures were identically repeated but staining was then carried out using a HRP-based solution (HRP-green solution; 42 Life Sciences, Cat#99056, Germany), staining type IIA fibers in green but leaving IIX fibers unstained. After dehydration of the cross sections (30 s 80% Ethanol, 30 s 100% Ethanol and 30 s Xylol), stained sections were air-dried covered with Entellan^®^ (Merck, Cat#107960, Darmstadt, Germany) and then covered with a glass slip. Five to 7 digital photos of each cross-section were captured in 20-fold magnification via a Zeiss KS-300 light microscope equipped with a digital CCD Camera (Sony, Japan). By applying the specific pixel/aspect ratio of the used 20X objectives (2.4 pixel per µm), the best fitting ellipse tool using the software ImageJ (National Institutes of Health, USA, RRID:SCR_003070) was applied to determine the inner borders of selected myofibers. Thirty-fivemyofibers per fiber type (type I and type II), time point and subject were analyzed for minor axis. As type IIX fibers could not be found in every subject, IIX fibers were excluded from myofiber diameter analysis. 

### 4.5. Rat Skeletal Muscle Histochemistry

Cross-sections (10 μm thick) were cut from extensor digitorum longus (EDL) and soleus rat muscles. The cross-sections were mounted on Vectabond (Vector Laboratories, Burlingame, CA, USA) coated slides and stored in −80 °C until further analysis. To determine the different fiber types in each rat skeletal muscle, images were captured from adenosinetriphosphatase (ATPase) stained cross-sections following the methods described in [64,65]. Digital pictures of cross-sections were captured using a 20× objective (DMRB microscope, Leica, Wetzlar, Germany) to finally differentiate between the four fiber types (type I, type IIA, type IIX, and type IIB [7].

### 4.6. Cell Culture

C2C12 muscle cells (ATCC, Cat# CRL-1772, RRID:CVCL_0188; Middlesex, UK; cells are regularly tested for contamination) were grown to confluency in growth medium containing Dulbecco’s modified Eagle’s medium (DMEM) (Gibco, Cat#31885, Waltham, MA, USA), containing 10% fetal bovine serum (Biowest, Cat#S181B, Nuaillé, France), 1% penicillin/streptomycin (Gibco, Cat#15140, Waltham, MA, USA) and 0.5% amphotericin B (Gibco, Cat#15290-026, Waltham, MA, USA) and incubated at 37 °C in humidified air with 5% CO2. Once 80% confluent, the medium was changed to differentiation medium consisting of DMEM supplemented with 2% horse serum (HyClone, Cat#10407223, Marlborough, MA, USA) and 1% penicillin/streptomycin. This medium was refreshed daily for 3 days before IGF-1 (100 ng/mL; recombinant human IGF-1, Peprotech, Cat#100-11, London, UK) was added for 24 h to induce hypertrophy in myotubes. 

### 4.7. RNA Isolation

From rat skeletal muscle, total RNA was extracted from the EDL and Soleus using the RiboPure kit (Applied Biosystems, Cat#AM1924, Foster City, CA, USA) according to the manufacturer’s instructions. RNA concentrations were determined in duplo by spectroscopy (ND-1000 spectrophotometer; Nanodrop Technologies, Wilmington, DE, USA). RNA purity was ensured by 260/280 ratio (range 2.00–2.11, mean 2.04). The muscle total RNA concentration was calculated on the basis of total RNA yield (μg) per weight (mg) of the analyzed sample.

For C2C12 cells, after washing cells with phosphate-buffered saline (PBS), cells were lysed in TRI reagent (Invitrogen, Cat#11312940, Carlsbad, CA, USA) and stored at −80 °C. RNA was isolated using RiboPure kit and converted to cDNA with high-capacity RNA to cDNA master mix (Applied Biosystems, Cat#4388950, Foster City, CA, USA). cDNA was diluted 10 times and stored at −20 °C.

### 4.8. Real-Time Quantitative PCR 

cDNA was analyzed using real-time quantitative PCR (see Table 1 for primer details). Experiments were conducted in duplicates. Concentration of the transcriptional target was detected with fluorescent SYBR Green Master Mix (Fischer Scientific, Cat#10556555, Pittsburgh, PA, USA). Transcriptional expressions of the target genes were referenced to 18 S housekeeping gene. Relative changes in gene expression were determined with the ΔCt method.

### 4.9. siRNA-Mediated Knockdown of Pkm, Pkm1 and Pkm2

In loss-of-function experiments, we knocked down *Pkm*, *Pkm1* and *Pkm2* in C2C12 myotubes using silencer RNA (Ambion, Carlsbad, CA, USA, see Table 2 for siRNA sequences). C2C12 myoblasts were grown and differentiated as described. On day 6, myotubes were transfected with siRNA targeted against *Pkm*, *Pkm1* or *Pkm2* using the liposome-mediated method (lipofectamine RNAiMAX, Invitrogen, Cat# 13778100, Carlsbad, CA, USA). As a negative control, a non-targeting silence RNA sequence (siControl) was used. siRNA was diluted in Opti-MEM medium and incubated for 5–10 min with lipofectamine mixture. RNA–lipofectamine complexes with a final concentration of 20 nM were added to each well. On day 7, the differentiated myotubes were treated with IGF-1 (100 ng/mL, Peprotech, Cat#100-11, London, UK) and harvested at day 8 (48 h post-transfection). 

### 4.10. Myotube Size Assay

Four photographs of each well containing differentiated myotubes were taken at 10x magnification after the 24 h treatment. Diameters were measured in 20–50 myotubes at 5 equidistant locations along the length of the cell using ImageJ (http://rsbweb.nih.gov/ij/; access 15/06/2019), National Institutes of Health, Bethesda, MD, USA; RRID:SCR_003070) and taking into account the pixel-to-aspect ratio. 

### 4.11. Statistical Analysis

Shapiro–Wilk tests were used to test for normal distribution. Data were then analyzed using unpaired *t*-test, two-way analysis of variance (ANOVA), or repeated measures ANOVA for normally distributed data. If data were not normally distributed, we used the Mann–Whitney U test or Friedman’s ANOVA for repeated measures. In the case of a significant ANOVA effect, a Bonferroni test was used to determine significant differences between conditions. Significance was set at *p* < 0.05. Data are presented as mean ± SEM with individual data points. Statistical analyses were performed using Prism 7.0 (GraphPad Prism, RRID:SCR_002798).

## Figures and Tables

**Table 1:** PCR primers.

Gene	Forward	Reverse
18 S rRNA	GTAACCCGTTGAACCCCATT	CCATCCAATCGGTAGTAGCG
*Pkm1*	CATGCAGCACCTGATAGCTC	TGAGGTCTGTGGAGTGACTG
*Pkm2*	CATGCAGCACCTGATTGCC	CCTCGAATAGCTCGCAAGTGG

**Table 2:** siRNA information.

Silenced Gene	Sense	Anti-Sense
*Pkm*	CCAUCAAGAAUGUCCGUGATT	UCACGGACAUUCUUGAUGGTC
*Pkm1*	GGCAGAGGCUGCCAUCUACTT	TTCCGUCUCCGACGGUAGAUG
*Pkm2*	GUGCGAGCCUCCAGUCACUTT	AGUGACUGGAGGCUCGCACTT
Control	AGUACUGCUUACGAUACGGTT	CCGUAUCGUAAGCAGUACUTT

## Supplementary Materials

The following are available online at https://www.mdpi.com/1422-0067/21/19/7062/s1, Figure S1: Resistance training induces muscle fiber hypertrophy, Figure S2: Resistance exercise regulates glycolytic enzyme abundance.
