# Supplementary figures and images for: PKM2 Determines Myofiber Hypertrophy In Vitro and Increases in Response to Resistance Exercise in Human Skeletal Muscle
Source: Int J Mol Sci. 2020 Sep 25;21(19):7062. doi: 10.3390/ijms21197062 (PMC7583908; doi:10.3390/ijms21197062)

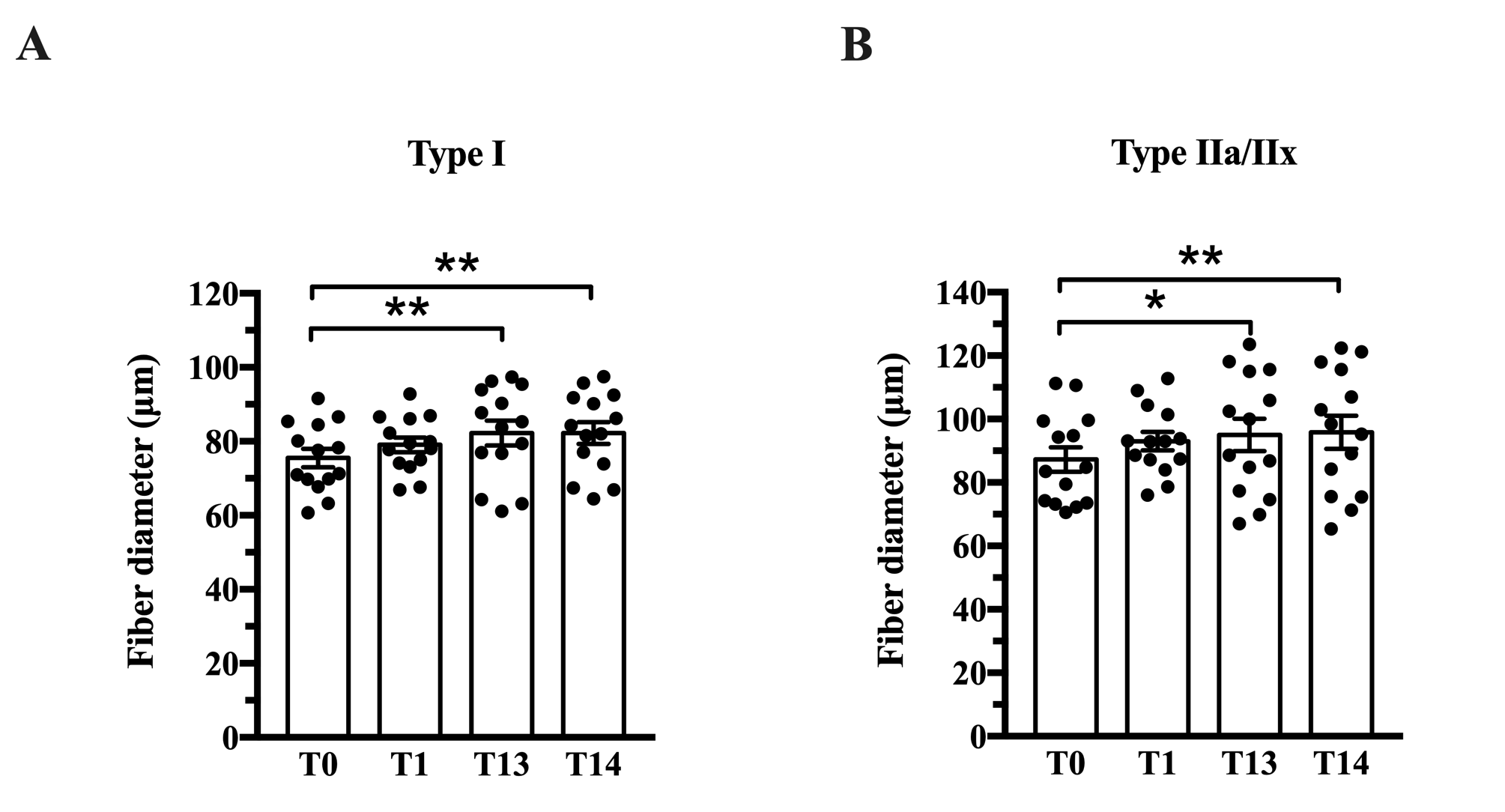

Supplement: Supplementary file 1 [file ijms-21-07062-s001.zip › Fig S1 - Resistance training induces hypertrophy in type I and type II fibers.tiff]

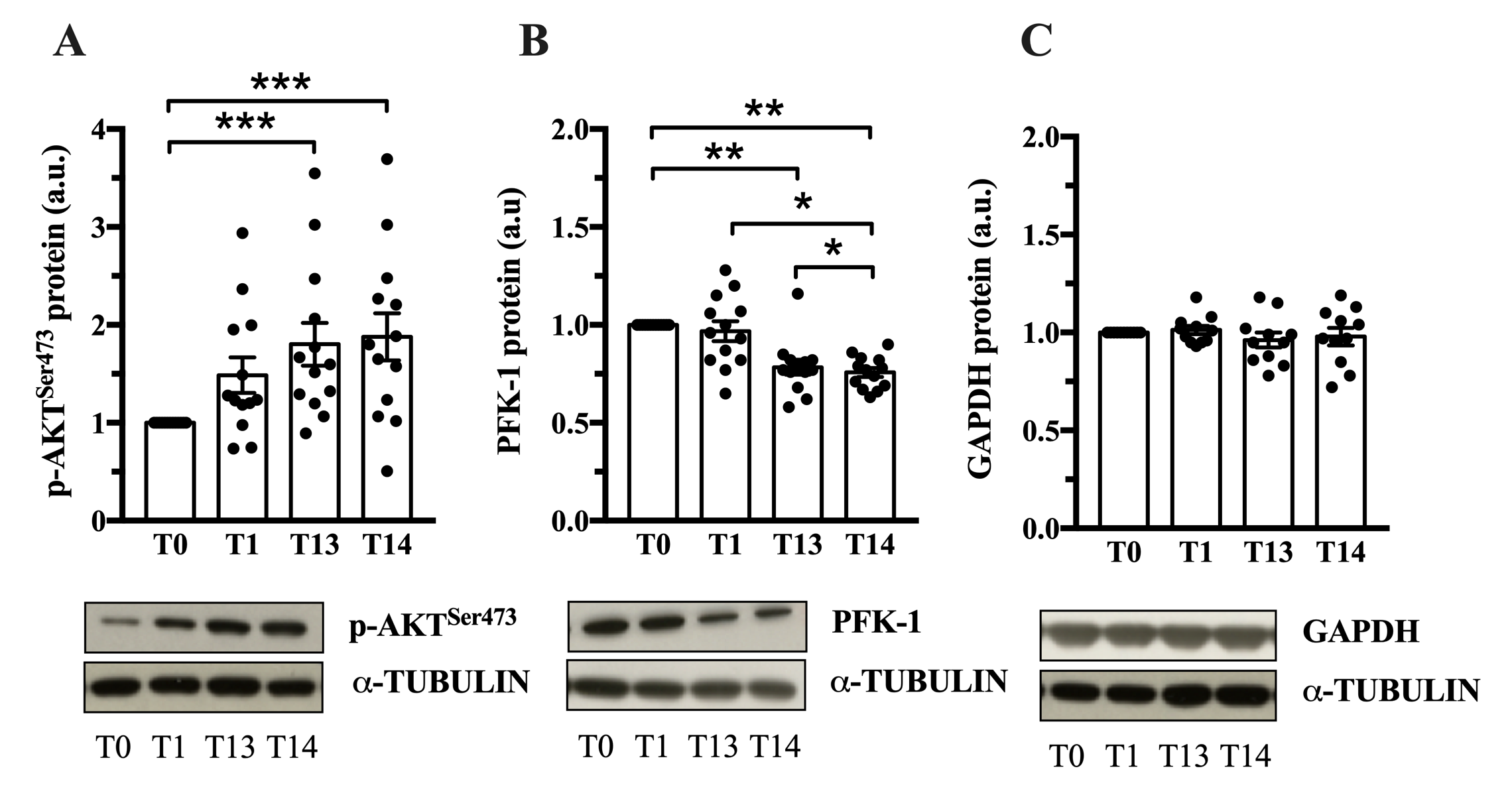

Supplement: Supplementary file 1 [file ijms-21-07062-s001.zip › Fig S2 - Resistance exercise regulates glycolytic protein abundance.tiff]
